# Rational Design of Supramolecular Fe and Mn Oxidation Catalysts

**DOI:** 10.1021/acs.accounts.6c00368

**Published:** 2026-07-21

**Authors:** Giorgio Olivo, Miquel Costas, Stefano Di Stefano

**Affiliations:** † Dipartimento di Chimica and Istituto CNR per i Sistemi Biologici (ISB-CNR), Sezione Meccanismi di Reazione, c/o Dipartimento di Chimica Università di Roma “La Sapienza”, P.le A.Moro 5, I-00185 Rome, Italy; ‡ Departament de Quimica Universitat de Girona Campus Montilivi, QBIS-Cat, Institut de Química Computacional i Catàlisi (IQCC), 17071 Girona, Catalonia, Spain

## Abstract

Modern organic synthesis aims to prepare the target molecule in a minimal number of steps. Hence, the efficiency, selectivity, and predictability of each of these steps need to be carefully optimized, even at a late stage of a synthetic route. This is particularly true in the case of aliphatic C–H functionalization, since differentiating one C–H bond among many others with similar properties in organic molecules often stands as an open challenge. Remote, inactivated C–H sites are especially difficult to target. Supramolecular catalysis represents one of the tools to overcome such problems. As occurs in enzymes, substrate binding to a supramolecular catalyst constrains their relative orientation, and the resulting preorganization can increase reactivity and unlock unusual selectivity in a rational and predictable manner, meeting late-stage functionalization requirements. In fact, supramolecular catalysis, which was initially oriented to the comprehension of the mechanisms of the involved reactions, is currently and rapidly evolving into a synthetically useful tool.

One decade ago, in 2017, we described Fe- and Mn-based supramolecular catalysts equipped with crown ether recognition sites able to efficiently catalyze the oxidation of inactivated C–H bonds in protonated primary amines. Substrate preorganization induced by recognition enables elusive oxidation at remote C8 and C9 sites in linear alkyl chains. Since then, a number of follow-up investigations allowed us to rationally elicit unnatural reactivity and selectivity in different bioinspired oxidation reactions, including predictable, late-stage C–H oxidation of steroids. In addition, an in-depth analysis of such oxidation processes provides a deep comprehension of the action mechanism of these supramolecular catalysts.

In this Account, we discuss the above investigations aiming to show (i) the rational approach to the design of a supramolecular oxidation catalyst, (ii) the experimental and theoretical tools that help understand and assess its mechanism of action, and (iii) its potential to address synthetic challenges. Eventually, a section that compares our approach to the one of other research groups illustrates the state of the art in supramolecular oxidation catalysis.

## Graphical abstract

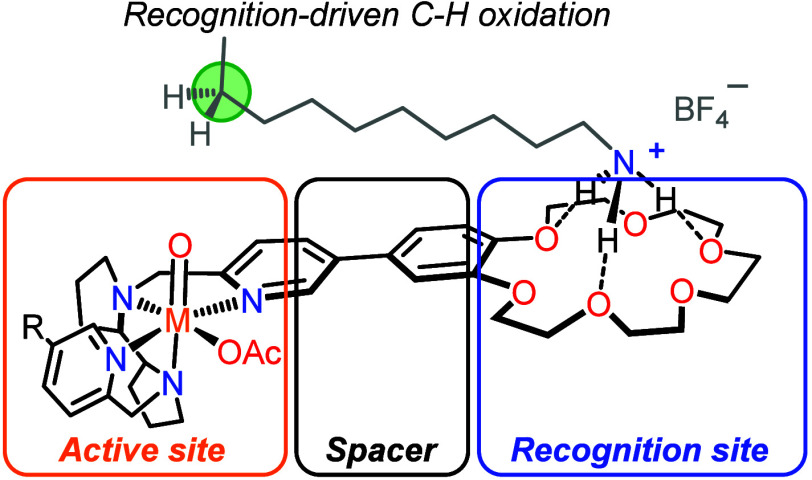

## Key References

-

Olivo, G. ; Farinelli, G. ; Barbieri, A. ; Lanzalunga, O. ; Di Stefano, S. ; Costas, M. Supramolecular Recognition Allows Remote, Site-Selective C–H Oxidation of Methylenic Sites in Linear Amines. Angew. Chem. Int. Ed. 2017, 56, 16347–16351 10.1002/anie.20170928029044918.^1^ This is our first work describing describing supramolecular Fe and Mn catalysts for remote C–H oxidation in linear alkyl chains guided by recognition.
-

Olivo, G. ; Capocasa, G. ; Ticconi, B. ; Lanzalunga, O. ; Di Stefano, S. ; Costas, M. Predictable Selectivity in Remote C–H Oxidation of Steroids via Analysis of Substrate Binding Mode. Angew. Chem. Int. Ed. 2020, 59, 12703–12708 10.1002/anie.20200307832337830.^2^ This work reports the application of our supramolecular system to late-stage C–H oxidation of steroid derivatives where selectivity can be predicted by NMR analysis of the adduct structure.
-

Vicens, L. ; Olivo, G. ; Costas, M. Remote Amino Acid Recognition Enables Effective Hydrogen Peroxide Activation at a Manganese Oxidation Catalyst. Angew. Chem. Int. Ed. 2022, 61, e202114932 10.1002/anie.202114932PMC930416634854188.^3^ In this report, remote recognition of an amino-acidic cofactor switches on productive H_2_O_2_ activation for asymmetric epoxidation at a Mn center, enabling the use of stoichiometric amounts of carboxylic acid cocatalyst.
-

Fagnano, A. ; Frateloreto, F. ; Paoloni, R. ; Sappino, C. ; Lanzalunga, O. ; Costas, M. ; Di Stefano, S. ; Olivo, G. Proximity Effects on the Reactivity of a Nonheme Iron­(IV) Oxo Complex in C–H Oxidation. Angew. Chem. Int. Ed. 2024, 63, e202401694 10.1002/anie.20240169438478739.^4^ This report analyzes and rationalizes how substrate recognition affects the rate of C–H oxidation and subsequent oxygen rebound within the supramolecular Fe catalyst–ammonium adduct.

## 1 Introduction

Selective C–H functionalization represents a longstanding problem in synthetic chemistry. Aliphatic C–H bonds are strong and nonpolar and hence poorly reactive. Moreover, organic molecules typically contain multiple C–H bonds with comparable physicochemical properties. Therefore, targeting a single C–H site stands as a formidable challenge that becomes even more demanding at late synthetic stages. Although precise functionalization methods following innate reactivity trends are being effectively developed, strategies to alter intrinsic selectivity and target indistinguishable sites remain scarce. Supramolecular catalysis represents one such strategy.

Supramolecular chemistry shifts the focus from “molecules composed by atoms” to “supramolecules composed by molecules” and held together by reversible interactions (i.e., hydrogen bonding, metal–ligand coordination, solvophobic, π-π, cation- or anion-π, etc.). Supramolecular catalysis was borne out of this concept with the aim of mimicking the catalytic activity of enzymes, regarded as ideal catalysts and sources of inspiration. Fast and reversible formation of a complex between the protein (the catalyst, Cat) and the substrate (Sub) has been recognized as a key contribution to the high efficiency and selectivity of enzymes already in 1913 by Michaelis and Menten.^5^ Such a fast pre-equilibrium relies on supramolecular interactions between Cat and Sub to bring in proximity and optimally orient (preorganize) the reactive functions for the ensuing reaction. Preassociation can dramatically accelerate the reaction, even when the uncatalyzed process is too slow to be practically detectable, and preorganization can provide excellent selectivity dictated by the adduct geometry. Finally, the reversibility of these interactions allows release of the product, leaving the enzyme free to enter a new catalytic cycle. Analogously, binding of molecules other than the substrate (cofactors) also modulates reactivity and selectivity, for instance by regulating the generation of the active species.

Supramolecular catalysts aim to replicate some aspects of this enzymatic preorganization to trigger, accelerate, and modulate artificial reactions. Substrate preassociation offers a unique chance to elicit unnatural reactivities and selectivities that are often difficult if not impossible to achieve otherwise. In addition, supramolecular recognition may confer a degree of substrate specificity, which is a common feature of enzymatic systems rarely displayed by artificial reagents. However, substrate specificity, which is often a limitation in biocatalysis, can be overcome with artificial supramolecular catalysts that generally display lower requirements for substrate recognition. The seminal works from the Lehn and Cram groups^6^ fueled an increasing interest in supramolecular catalysis that remains vibrant up to this day. Initial works mainly focused on reaction mechanisms to understand and rationalize the observed improvements in activity and selectivity.^7–9^ Later, supramolecular catalysts have been applied to address open challenges in synthetic chemistry, spanning from control of selectivity to unlocking new reactivity and achieving temporal regulations, as discussed in several general review articles.^9,10^

Site-selective oxidation of aliphatic C–H bonds in organic molecules represents one of the standing problems in organic chemistry that has no simple solutions. In the last decades, the design of bioinspired iron and manganese catalysts that generate enzyme-like oxidants (high-valent metal-oxo) powerful enough to cleave C–H bonds and display chemo and site selectivity has emerged as an effective strategy for sustainable and selective C–H oxidation.^11,12^ In the following sections, we will discuss the rational design of supramolecular versions of these catalysts pioneered by us^1–4,13–15^ and others.^16–18^ First, we will analyze how recognition affects substrate oxidation, carefully taking into account the thermodynamic and kinetic parameters at play (the reaction mechanism of a supramolecular catalyst).^4,15^ Then we will discuss how these systems can achieve unnatural reactivity^3,4^ and selectivity^1,2,13^ in reactions of synthetic relevance in light of the acquired knowledge. Eventually, we will put these results in the context of related supramolecular Fe and Mn oxidation catalysts developed by other groups (Section 7).^16–20^

## 2 Subunit Design of Supramolecular Oxidation Catalysts

Our supramolecular catalyst consists of three main units (Figure 1A): (i) an active site that catalyzes the oxidation, (ii) a recognition site deputed to the selective binding and preorganization of the substrate (or the cofactor), and (iii) a spacer, which connects the former ones.

**Figure 1:**
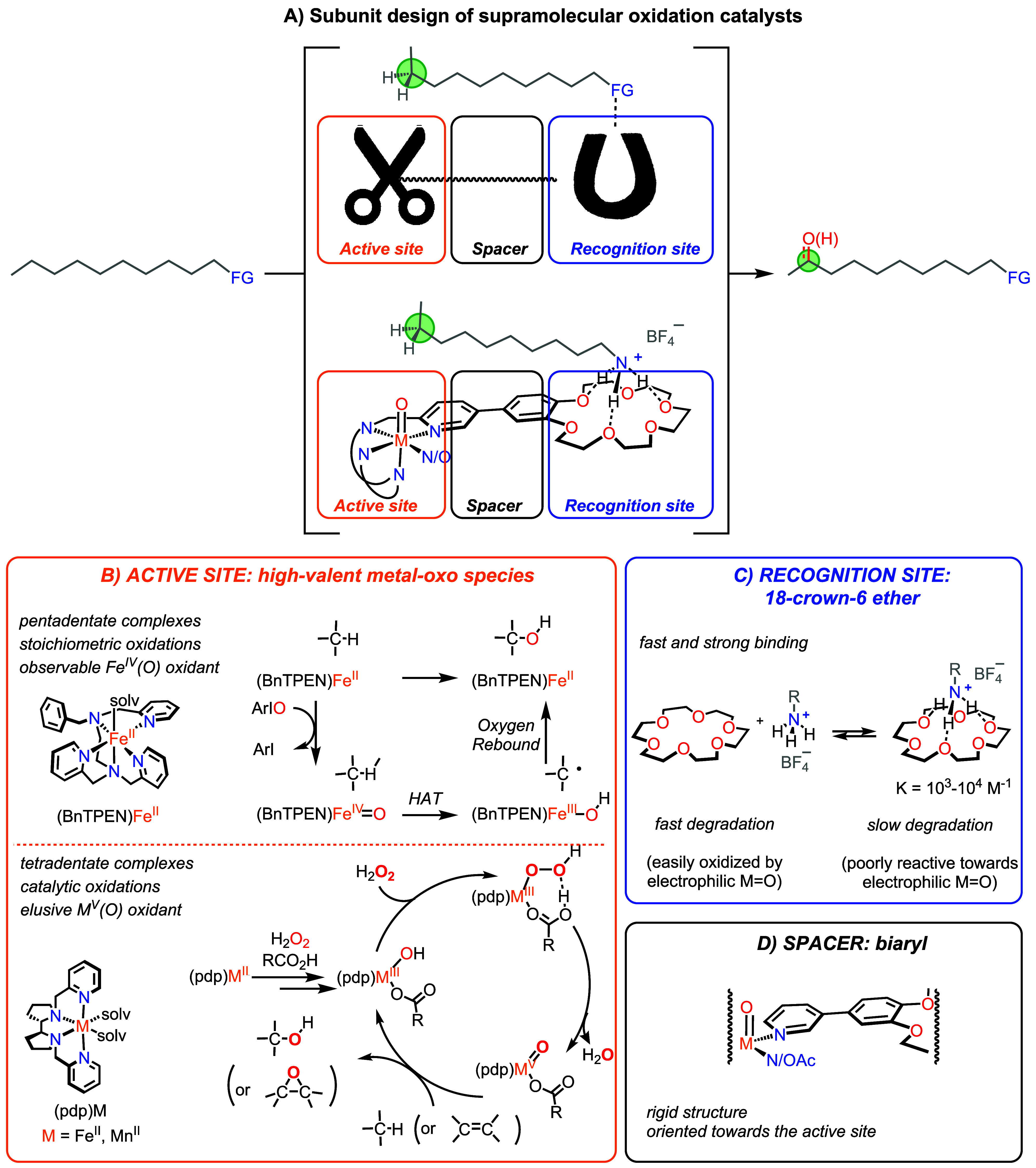
(A) Subunit design of a supramolecular catalyst. (B) High-valent metal-oxo active sites. (C) 18-Crown-6 recognition site. (D) Biaryl spacer.

### 2.1 Active Site

The active site is responsible for the reactivity. As such, it must effectively perform the desired reaction (C–H oxidation) throughout multiple catalytic cycles. The reaction should exclusively occur at the catalytic center to avoid background processes (i.e., the catalyzed reaction should fully outcompete the uncatalyzed one; ideally, the reaction should not occur without a catalyst). Eventually, neither the active site nor the substrate or reactants should interfere with the recognition process.

In our case (Figure 1B), the active site consists of a high-valent metal-oxo complex (Fe or Mn) competent for biomimetic oxidations, such as the hydroxylation of inactivated C–H bonds (via hydrogen atom transfer/oxygen rebound).^12^ Careful choice of the Fe^II^ or Mn^II^ complex structure (defined by its supporting penta- or tetradentate aminopyridine ligand) and of the oxidant (stoichiometric iodosyl benzene ArI=O derivatives^21^ or excess H_2_O_2_ ^22^) allows us to switch from stoichiometric reactions functional for a mechanism investigation to catalytic, synthetically useful oxidations processes. Pentadentate complexes such as (BnTPEN)Fe react with ArI=O to generate isolable (BnTPEN)­Fe^IV^(O) complexes, competent for slow, stoichiometric C–H oxidation.^21,23^ On the other hand, tetradentate complexes such as (pdp)Fe and (pdp)Mn catalytically activate H_2_O_2_ with the aid of carboxylic acids (typically acetic acid, AcOH)^24^ to generate powerful (pdp)­M^V^(O)­(OAc) (M = Fe, Mn) oxidants,^25^ competent for fast, catalytic oxidations.

### 2.2 Recognition Site

The recognition site binds and preorganizes the substrate (and/or the cofactor). In enzymes, both the peptide backbone and the lateral amino acid side chains are exploited to establish supramolecular interactions with the substrate (or the cofactor). The reversibility of these interactions coupled with a selective recognition of the substrate over the product allows catalytic turnover and minimizes product inhibition. Replication of this sophisticated machinery in artificial catalysts is far from straightforward. A typical yet not optimal solution simplifies the problem and uses supramolecular receptors to bind both the substrate and the product with similar binding constants, relying on the reversibility of the binding to enable catalytic turnover.

In our case, we resorted to 18-crown-6 ethers as recognition sites for the selective binding of molecules endowed with a primary ammonium head (Figure 1C).^26^ The multiple hydrogen bonding interaction secures high enough association constants (10^3^–10^4^ M^–1^ in wet acetonitrile) even when the crown ether is attached to a dicationic M^II^ complex as depicted in Figure 1A. Hence, under typical oxidation conditions (substrate ≈ 0.1 M, catalyst ≈ 0.001 M), most of the catalyst (>99%) is bound to the ammonium substrate. In addition, binding of positively charged ammonium ion polarly deactivates and thus prevents oxidation of C–H bonds of the crown ether by electrophilic metal-oxo species, increasing catalyst robustness. Exchange of primary ammonium ions is fast on NMR time scale (milliseconds), as confirmed by the single set of ^1^H NMR signals observed for both the receptor and the substrate, compatible with catalytic turnover. Eventually, cationic ammonium ions cannot interact with the metal center and lead to its deactivation, which was a significant problem for other Lewis bases receptors tested, such as urea motifs.

### 2.3 Spacer

The structure and orientation of the spacer determine the efficiency of a supramolecular catalyst (Figure 1D). The best solution is represented by a rigid motif, which reduces conformational freedom within the Cat–Sub adduct and ideally does not generate significant strain when closing the pseudocyclic transition state of the catalyzed reaction (*vide infra*). In other words, the spacer strongly contributes to preorganization, somehow orienting the substrate toward the active site. Any misalignment at this stage translates to losses of efficiency and selectivity. Eventually, the spacer should not influence the reaction that occurs at the active site.

We identified a good compromise in the biaryl linkage, which is rigid and synthetically easy to install via cross coupling.^27^ It also allows good electronic isolation between the catalytic and recognition sites, simplifying kinetic analyses. The spacer is attached to the β position(s) of pyridine rings in the ligand not to affect metal coordination and the ensuing H_2_O_2_ activation,^12^ while simultaneously orienting the bound substrate toward the metal-oxo center.

## 3 Consequences of Recognition: Proximity Enhances Reactivity

### 3.1 Accelerating the Oxidation

Recognition positions the substrate close to the reactive metal-oxo unit. The ensuing proximity between the reactive functions accelerates the oxidation, which becomes pseudointramolecular.

From a mechanistic perspective, C–H hydroxylation involves two steps, (i) a rate-determining Hydrogen Atom Transfer (HAT) from the C–H bond to the metal-oxo and (ii) and a fast recombination of the carbon radical with the hydroxide ligand, named Oxygen Rebound.^28^ Kinetic analysis is limited to the first, rate determining HAT step and experimentally involves monitoring the metal-oxo decay in the presence of decyl-ammonium substrate (DecNH_3_^+^) with two complexes that only differ for the presence (or absence) of the receptor. Such analysis is nearly impossible with catalytically relevant (pdp)­Fe^V^(O) species (<5% accumulation reported at −80 °C^29^) but becomes feasible with a structurally similar (BnTPEN)­Fe^IV^(O) species (>90% accumulation at 25 °C), which was selected for the kinetic investigation. Oxidation of DecNH_3_^+^ is rather slow with the model (BnTPEN)­Fe^IV^(O) (*k*_2_ = 2.9 × 10^–4^ M^–1^ s^–1^), as expected, but accelerates with the supramolecular complex (^CR^BnTPEN)­Fe^IV^(O) (1–2 orders of magnitude increase of initial rates). More importantly, in the latter case, a saturation profile of initial rates vs substrate concentration is observed. Such profile can be fitted with the Michaelis Menten model to give *k*_cat_ = 2.0 × 10^–4^ s^–1^ and *K*_M_ = 8.0 × 10^–4^ M, with an apparent association constant (*K*_M_^–1^ = 1.2 × 10^3^ M^–1^) well-comparable to the binding constant obtained by ^1^H NMR titrating the Fe^II^ precursor (^CR^BnTPEN)­Fe^II^ with DecNH_3_^+^ (1.7 × 10^3^ M^–1^). The observation of a saturation behavior, the acceleration of initial rates and the alteration of site-selectivity (*vide infra*) confirm that the oxidation occurs within the Cat–Sub adduct (pseudointramolecular reaction).

### 3.2 Rationalizing Rate Enhancements

The proximity between the catalytically active species and the reacting site of the substrate is a main contribution to enzymatic efficiency. Once the enzyme binds its substrate, the reaction occurs within this adduct, becoming pseudointramolecular, and a (large) increase of reaction rates is generally observed. The same factors are at play with supramolecular catalysts. The Effective Molarity concept (EM = *k*_intra_ */k*_inter_) was introduced to quantify the increase of reactivity^8,30,31^ related to (pseudo) intramolecularity. In the case of supramolecular catalysts, EM is defined as the *k*_cat_/*k*_2_ ratio, where *k*_cat_ and *k*_2_ are the rate constants for the pseudointramolecular reaction and its intermolecular model reaction, respectively, with the caveat that both processes must proceed with the same mechanism. The higher the EM, the more facile the approach of the substrate reacting site to the catalytic center during the conversion of the acyclic Cat–Sub adduct into a pseudocyclic transition state. More in detail, EM can be considered as the product of two contributions, EM = EM_*H*_ EM_*S*_, one enthalpic and one entropic in nature. EM_*H*_ quantifies the strain energy developed during the formation of the pseudocyclic transition state (the higher such strain, the more difficult the approach, EM_*H*_ ≪ 1). The entropic one (EM_*S*_) is correlated to the number of rotatable bonds that have to be frozen to close such pseudocyclic transition state (the higher the number of rotors to freeze, the lower the EM_*S*_ value). Theoretically speaking, the maximum value of EM is reached when no strain energy is developed at transition state level (EM_*H*_ = 1 and EM = EM_*S*_), i.e., the catalyzed pseudointramolecular process exploits all the advantage offered by the intramolecularity. A list of EM_*S*_ values as a function of the number of frozen rotors has been made combining an empirical treatment for smaller rings with Jacobson and Stockmayer’s theory applied to irreversible macrocyclization for larger rings.^8,30,32^

In our case, pseudointramolecular oxidation of DecNH_3_^+^ by (^CR^BnTPEN)­Fe^IV^(O) (with its *k*_cat_) is compared to its ideal intermolecular model counterpart, i.e., intermolecular oxidation by (BnTPEN)­Fe^IV^(O) (with its *k*_2_). Product analysis weighs the contribution of each DecNH_3_^+^ methylenic site (*k*_cat_ (sel at site) and *k*_2_ (sel at site)) to the overall oxidation (total kinetic constant *k*_cat_ and *k*_2_, see eqs 1 and 2).^4^ Analogously, each methylene contributes to the overall EM by a factor given by the ratios EM_site_ = *k*_cat_ (sel at site)/*k*_2_ (sel at site) (Figure 2B).

1 $$\Sigma_{site}k_{cat}\;(sel\;at\;site)=k_{cat}$$

2 $$\Sigma_{site}k_{2}\;\left(sel\;at\;site\right)=k_{2}$$

**Figure 2:**
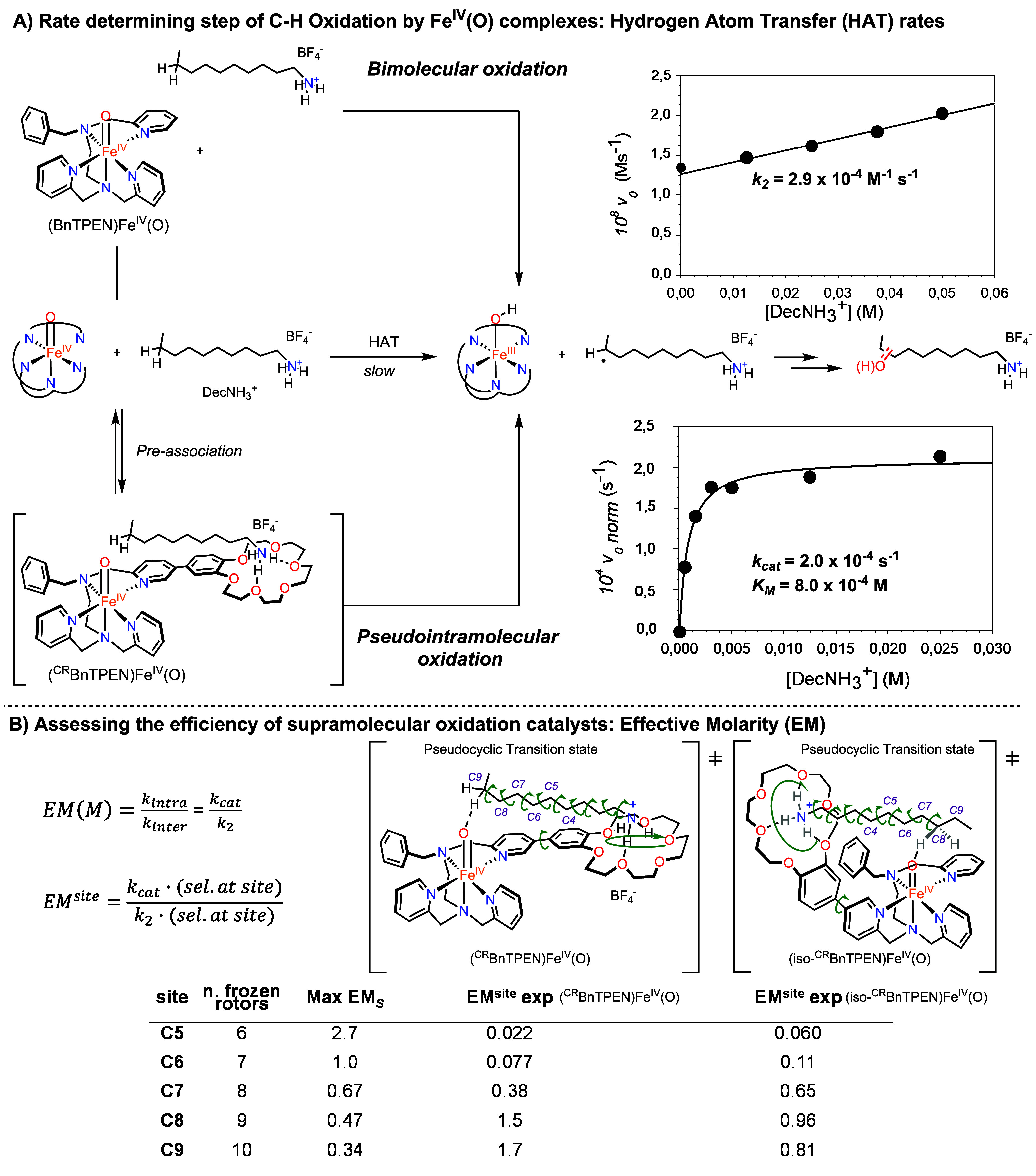
(A) DecNH_3_^+^oxidation by (BnTPEN)­Fe^IV^(O) (bimolecular) or (^CR^BnTPEN)­Fe^IV^(O) (pseudointramolecular). (B) Measure of the efficiency of supramolecular oxidation catalysts by EM analysis.

With (^CR^BnTPEN)­Fe^IV^(O), experimental EM­(site)­s are well below theoretical EM*_S_* for oxidation at C5 and C6 and only slightly for C7 sites, pointing to significant strain developed during the approach of the two reactive functions (EM_*H*_ ≪ 1 for C5 and C6, and EM_*H*_ < 1 for C7). In contrast, they well compare to (or are slightly higher than)^33^ theoretical EM*_S_* values for C8 and C9 sites, indicating that no relevant strain is developed at transition state level (EM_*H*_ ≈ 1).^4^ In other words, supramolecular catalyst (^CR^BnTPEN)­Fe^IV^(O) operates at the maximal possible efficiency when promoting C8 and C9 oxidation. A similar complex where the crown ether receptor is located at an analogous distance but with a different orientation (iso-(Bn^CR^TPEN)­Fe^IV^(O), with perpendicular vs parallel recognition and catalytic sites (Figure 2B), affords comparable or slightly lower EM values, with only little variations in reactivity and site-selectivity at each site.^15^

### 3.3 Beyond the Rate-Determining Step: Modulating Oxygen Rebound Efficiency

Proximity increases the efficiency not only of the initial HAT but also of the subsequent oxygen rebound. Experimentally, analysis of this step implies isotopic ^18^O labeling of the metal-oxo to distinguish alcohol products formed via hydroxyl rebound (^18^O-labeled) from those obtained via competing radical diffusion and dioxygen trapping (unlabeled). With the model (BnTPEN)­Fe^IV^(O) system, comparable oxygen rebound is observed across all methylenes (45–48%, the remainder derives from O_2_, Figure 3). With our supramolecular system, the oxygen rebound is analogous to the model for sites disfavored by recognition (44% at C6) and then increases its efficiency on methylenes favored by recognition up to remarkable 82% and 87% at C8 and C9 sites.^4^ These observations indicate that proximity of the substrate to the iron center, induced by supramolecular recognition, favors oxygen rebound, providing validation for the biochemical hypothesis that modulation of rebound efficiency (key to inducing elusive processes different from hydroxylation) depends on proximity.^34^

**Figure 3:**
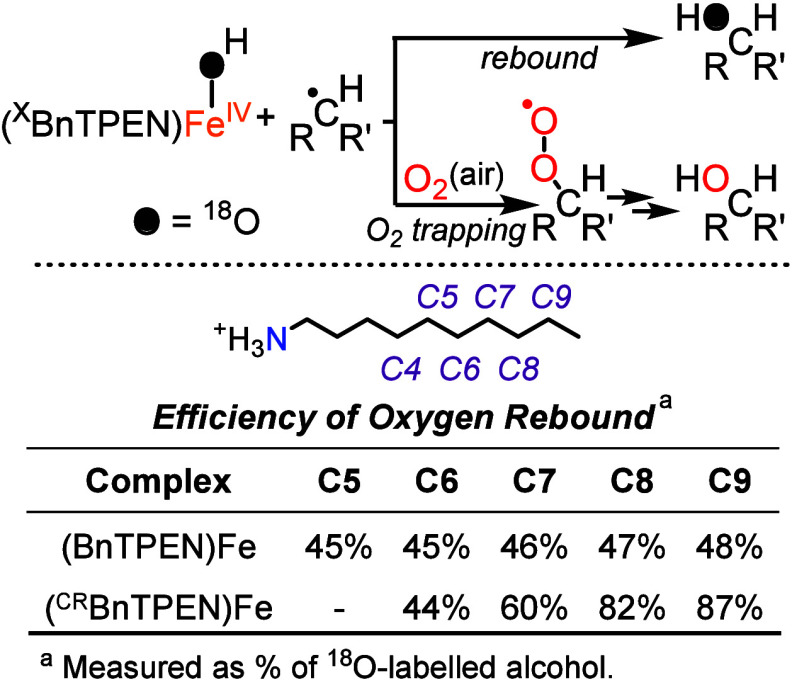
Modulation of oxygen rebound efficiency by proximity.

### 3.4 Exploiting Rate Enhancement: Substrate-Selective Oxidations

Once recognized, bound substrates are oxidized faster than unbound ones, even when they are intrinsically less reactive. In other words, recognition can alter the natural relative reactivities of two molecules in competing oxidations. This feature lies at the heart of the specificity of natural enzymes but remains elusive for conventional catalysts.

To explore this feature, we focused on catalytic, synthetically useful oxidations with tetradentate (pdp)­Mn catalysts and H_2_O_2_. Competitive oxidation of undecyl ammonium, with secondary C–H bonds, and menthyl acetate (1:1 mixture), containing weaker, tertiary C–H bonds results in the preferential oxidation of the latter (∼20% ammonium selectivity, 1:4 ratio; Figure 4A) with the model (pdp)Mn catalyst. In contrast, the supramolecular catalyst (^CR^pdp)Mn reverses such selectivity, leading to almost exclusive oxidation of the former (>95% selectivity, >50:1 ratio).^13^ Such reactivity enhancement is large enough to preferentially oxidize nonactivated methylenes of the ammonium even in the presence of activated, benzylic C–H bonds. Even in a pool of five competing substrates, the supramolecular catalyst effectively “fishes out” the ammonium (selectivity amplified from 3% to 77%, Figure 4A).

**Figure 4:**
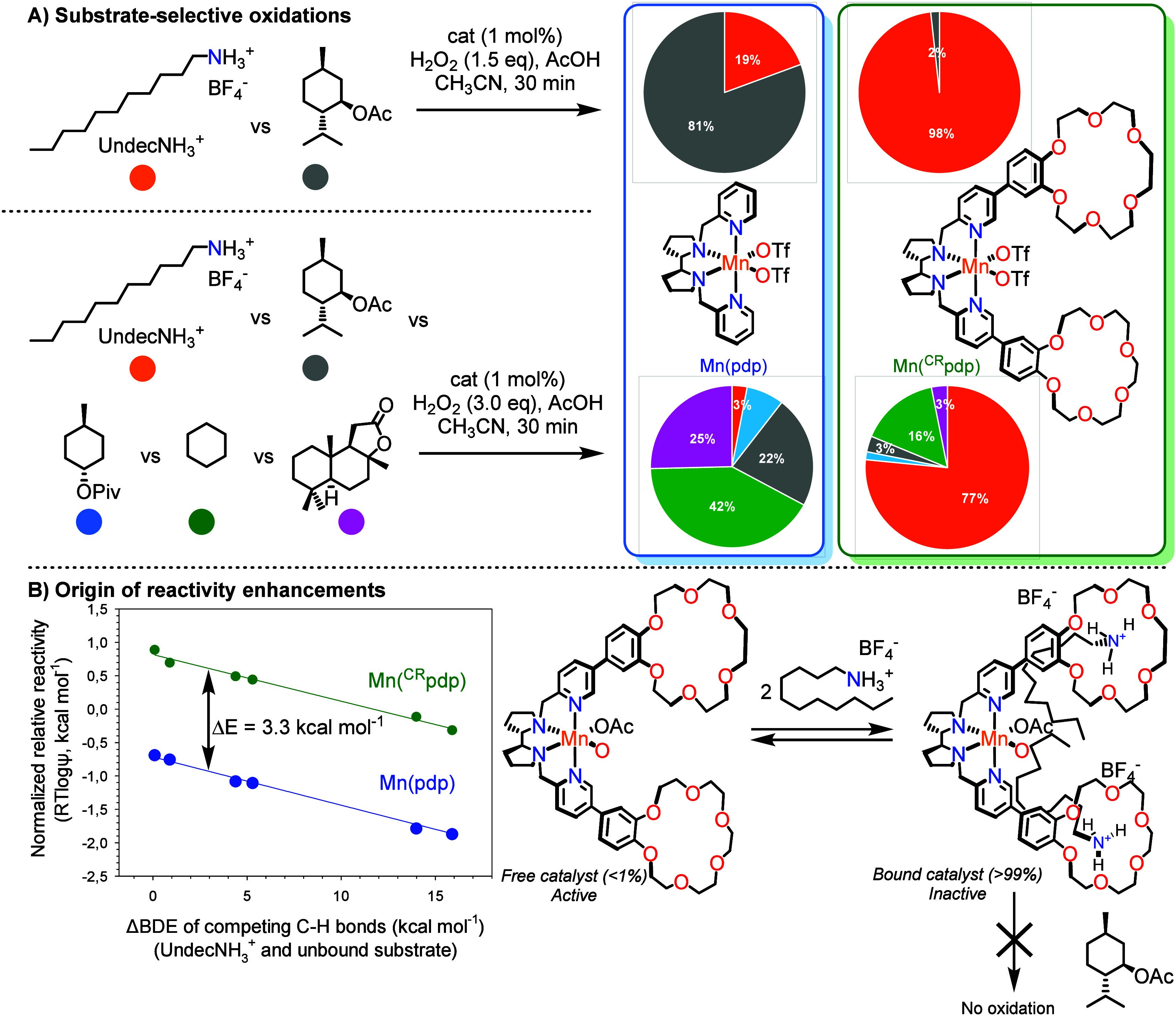
(A) Selective oxidation of bound over unbound substrates. (B) Analysis of proximity-induced rate enhancement.

Such preference for bound substrates, measured as normalized relative reactivity at the most oxidizable C–H bonds,^13^ can be plotted against the difference in competing C–H bond strength (Figure 4B). The experimental points dispose on two straight, parallel lines for the two catalysts (model (pdp)Mn and supramolecular (^CR^pdp)­Mn). Such disposition implies that (i) both reactions occur with the same mechanism, with a comparable dependence on C–H bond strength; (ii) the energy difference between the two lines quantifies the reactivity enhancement due to proximity (normalized per C–H bond and measured at recognition-favored sites), which results in a 3.3 kcal mol^–1^ difference (EM ≈ 250 M at 298 K).^35^

Such reactivity enhancement is much higher than that obtained in direct kinetic measurements with the related (^CR^BnTPEN)­Fe^IV^(O) complex (EM ≈ 250 M vs EM ≈ 1–1.7 M, respectively, always referred to C8 and C9 sites). Although the differences in the systems (tetra- vs penta-dentate ligand, Mn^V^(O) vs Fe^IV^(O) active species, one vs two receptors) contribute to this discrepancy, they cannot adequately explain such a large difference, indicating that some other effects are at play. As proposed by Crabtree et al.,^36^ when undecyl ammonium binds to the supramolecular catalyst, the reactive metal-oxo active site becomes sterically less accessible to other molecules freely diffusing in solution (i.e., the competing substrate). In other words, the bound ammonium acts both as a substrate and as an inhibitor for the catalyst, but only toward unbound molecules (Figure 4B). Hypothesizing that the catalyst:ammonium adduct does not oxidize unbound substrates at all, the active fraction of the catalyst under reaction conditions is <1/100 based on a 10^4^ M^–1^ association constant (see above). This is fully consistent with the 2 orders of magnitude difference in reactivity observed. Hence, the efficiency of the catalyst derives both from reactivity enhancement for bound substrates and reactivity reduction for unbound ones.

## 4 Consequences of Recognition: Control of Selectivity

### 4.1 Geometric Selectivity for Remote Sites

Supramolecular recognition fixes the relative orientation between the bound (ammonium) substrate and the active site, placing some positions (C–H bonds) in proximity to the reactive function (metal-oxo) and other ones further away. The sites close to the metal-oxo unit react faster than the others and are preferentially oxidized. Hence, purely geometric considerations allow us to rationally modulate and predict selectivity.

Initially, the oxidation of flexible, linear alkyl chains of different lengths bearing a primary ammonium head was analyzed.^1^ In these compounds, electronic effects deactivate the C–H bonds close to the cationic head (C1–C5, toward electrophilic metal-oxo species)^37^ and the terminal CH_3_ (stronger primary C–H bonds), while steric accessibility slightly increases the reactivity of the last (ω-1) methylene. The remaining internal CH_2_ have comparable properties and display similar reactivity. In fact, oxidation of these molecules with the model Mn­(pdp) catalyst perfectly reflects such scenario (Figure 5, left).

**Figure 5:**
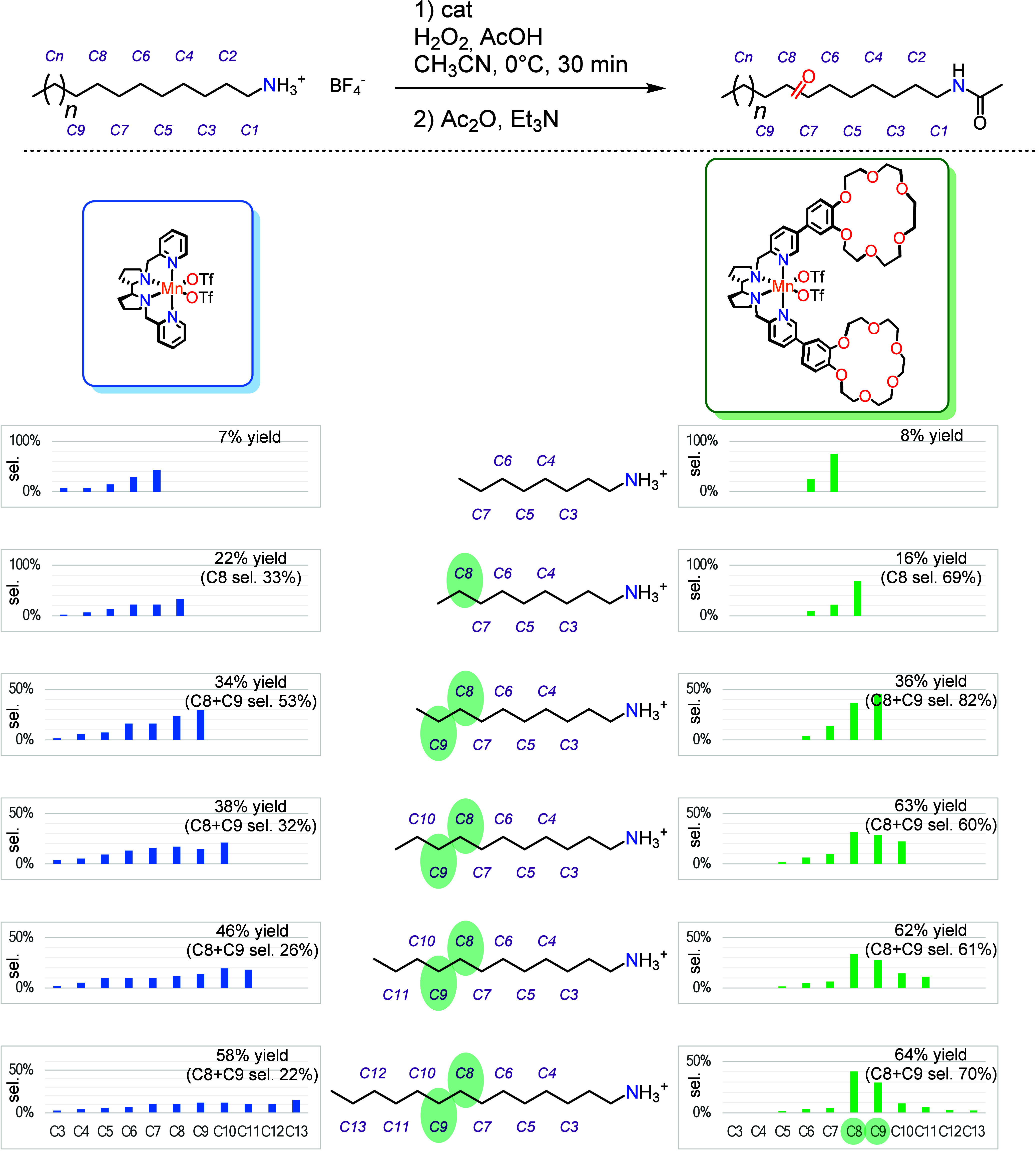
Recognition-controlled site-selective oxidation of alkyl ammoniums.

In contrast, the use of supramolecular catalyst Mn­(^CR^pdp) (or Fe­(^CR^pdp)) leads to an unnatural bell-shaped distribution of oxidation products, with the maximum invariably centered at remote C8 and C9 methylenes, irrespective of the chain length (Figure 5, right column).^1^ The selectivity for these sites increases from 22–35% with Mn­(pdp)^38^ to 60–82% with (^CR^pdp)­Mn, providing an effective strategy to differentiate methylenes otherwise undistinguishable, also in light of previous reports.^39^ Such a preference stems from substrate recognition and preorganization as confirmed by a series of control experiments where the bell-shaped product distribution is lost, restoring the natural selectivity pattern of Mn­(pdp), whenever the receptor is incapable of acting as a substrate recognition unit.^1^

### 4.2 Improving Selectivity

Further improvement of such a remote selectivity is not trivial. EM analysis on related (^CR^BnTPEN)Fe indicates that the supramolecular catalyst already operates at its maximum efficiency (*vide supra*),^4^ and hence any additional increase requires reduction of the conformational freedom within the Cat–Sub adduct (decrease of the number of free rotatable bonds to be frozen).

Simultaneous binding of the ammonium heads of a diammonium chain to both two crown ether receptors can increase rigidity (Figure 6A).^14^ Protonated 1,14-tetradecyl diamine is long enough to bind both two crown ether receptors in (^CR^pdp)­Mn, as confirmed by NOESY analysis. The two-point recognition fixes the conformation of the alkyl chain, which is not free anymore to coil and bend but is forced into a straight, quite rigid conformation. As a result, the oxidation selectively occurs on the central methylene sites (C6 and C7). The selectivity for their oxidation increases from 62% up to 88%, which can be improved up to 92% by fine-tuning of the carboxylic acid cocatalyst (which is bound to the oxidizing Mn^V^=O species).

**Figure 6:**
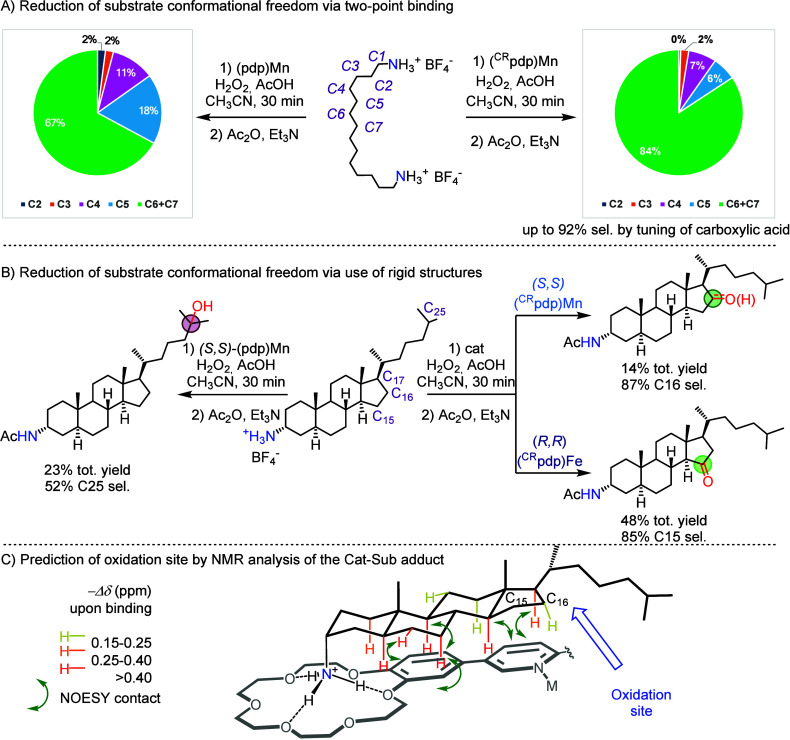
Improving site-selectivity by reducing substrate conformational freedom. (A) Two-point binding. (B) Rigid substrates. (C) Prediction of oxidation site.

Conformational freedom can be also restricted using rigid substrates, such as the flat core of steroids (3α-amino-androstane derivatives, Figure 6B).^2^ These substrates are optimal platforms for late-stage C–H functionalization, as they combine medicinal relevance to a complex structure with multiple C–H bonds and other reactive groups. Not surprisingly, oxidation of this compound with the model Mn­(pdp) catalyst leads to a complex mixture of oxygenated products, with a slight preference for the accessible tertiary C25–H bond on the lateral chain (up to 23% overall yield, 52% selectivity, with conventional catalysts). Our supramolecular systems alter natural product distribution, suppressing hydroxylation on the lateral chain (<5% C25 selectivity) and amplifying oxidation at the D-ring (>90% combined selectivity for C15, C16, and C17 sites). Modulation of catalyst chirality and metal fine-tunes the selectivity, with (*S*,*S*)-(^CR^pdp)Mn favoring hydroxylation at C16 (13% yield, 87% selectivity) and (*R*,*R*)-(^CR^pdp)­Fe) preferring oxidation at C15 site (41% yield, 85% selectivity).^40^ Analogous results are obtained with other androstane derivatives. Interestingly, the sites oxidized in the steroid scaffold are located 8 or 9 carbon atoms away from the ammonium head, and their proximity to the metal center can be confirmed by NMR analysis prior to the oxidation (recognition-induced shifts, intermolecular NOESY contacts in an analogous Zn complex, see Figure 6C).

## 5 Consequences of Recognition: Shifting the Focus from the Substrate

Binding of molecules other than the substrate offers a chance to modulate reactivity and selectivity and even unlock precluded reaction pathways. For instance, in iron oxygenases, formation of the active iron-oxo requires a single acidic residue in proximity to the metal. This residue delivers a proton to the distal O atom of the Fe^III^–OO­(H) intermediate (formed upon O_2_ binding), facilitating O–O bond heterolysis. In bioinspired catalysts, such a proton is delivered by an external carboxylic acid that binds to the metal center in a *cis*-labile coordination site and regenerated by H_2_O_2_.^22^ However, this external acid needs to be added in large excesses, typically >8000 equiv for Mn catalysts.^12^

Supramolecular recognition offers a way to place such a single acidic function close to the metal center, replicating the efficiency of enzymes. Once a protonated ω-amino acid of appropriate length binds to the crown ether receptor, the carboxylic acid function is brought in proximity to the metal center, enabling enzyme-like H_2_O_2_ activation at nearly stoichiometric acid loadings (Figure 7).^3^ In fact, 1.2 equiv of this acid with respect to the Mn catalyst do not allow olefin epoxidation with model Mn systems (0–3% yield) but are enough with a supramolecular catalyst (87% yield) even at −40 °C. Again, control experiments highlight that whenever remote ammonium recognition is precluded, catalytic activity drops (0–6% yield). Furthermore, selection of the amino acid lateral chain modulates the structure the oxidizing species, fine-tuning activity (epoxide yield) and chirality induction (enantioselectivity), with optimal cocatalysts composed of α-amino acids with rigid, bulky lateral chains (i.e., tert-leucine) coupled to a linear 6-amino hexanoyl unit (up to 65% yield, 67% ee in the epoxidation of a flexible olefin).

**Figure 7:**
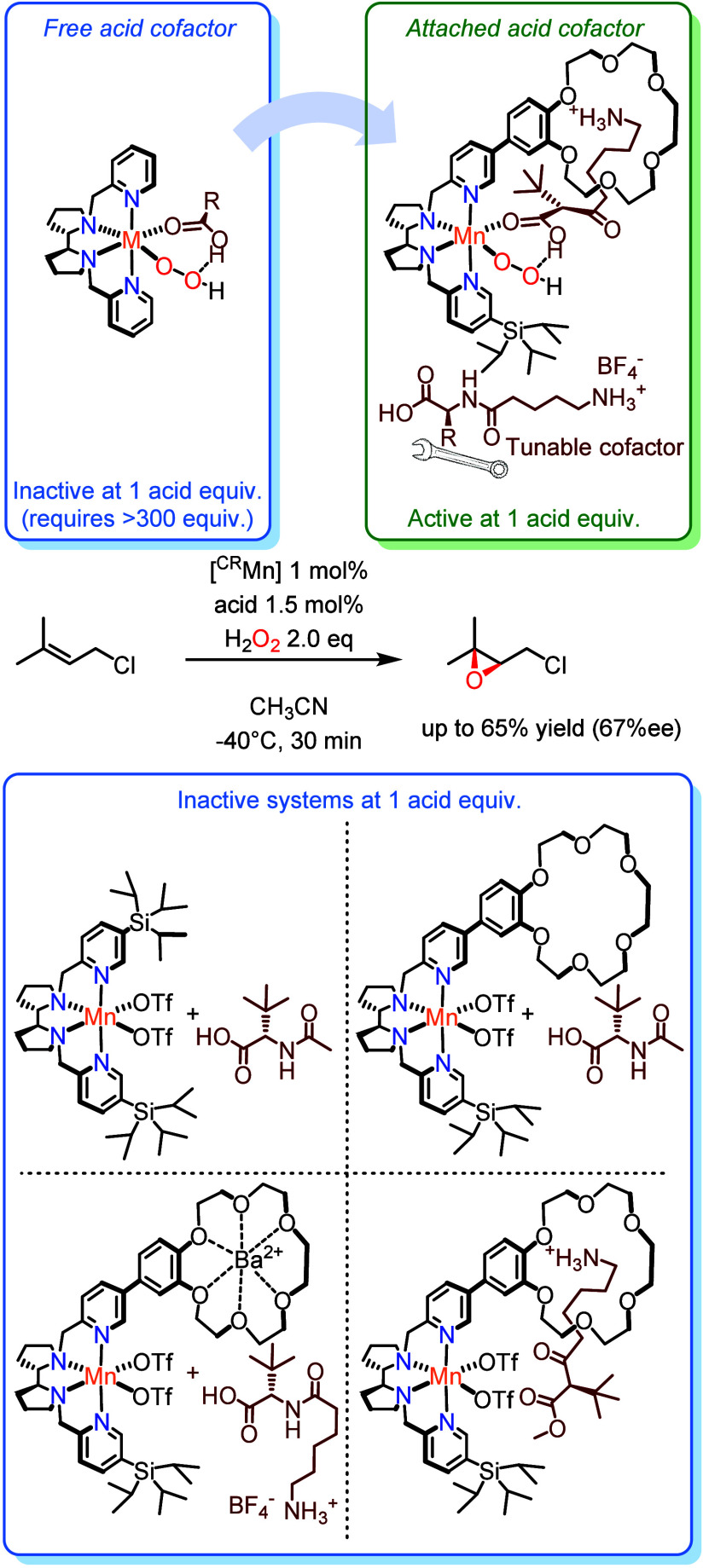
Cofactor (carboxylic acid) binding enables effective epoxidation with H_2_O_2_.

## 6 State of the Art: Other Supramolecular Oxidation Catalysts

Substrate preorganization inside natural oxygenases has inspired the design of artificial supramolecular catalysts ever since the pioneering reports of Breslow in the ’90s (Figure 8).^41^ These works demonstrated the feasibility of selective C–H hydroxylation by fixing substrate positioning via simultaneous binding of two ^*t*^Bu-phenyl groups in cyclodextrins^42^ or carboxylic acid dimerization in aprotic, anhydrous solvents.^20^ Unfortunately, the sophistication of such supramolecular recognition, which requires double binding, complementary and rigid substrates and/or well-controlled conditions, has limited their applicability to few substrate structures, and few quantitative studies were performed.

**Figure 8:**
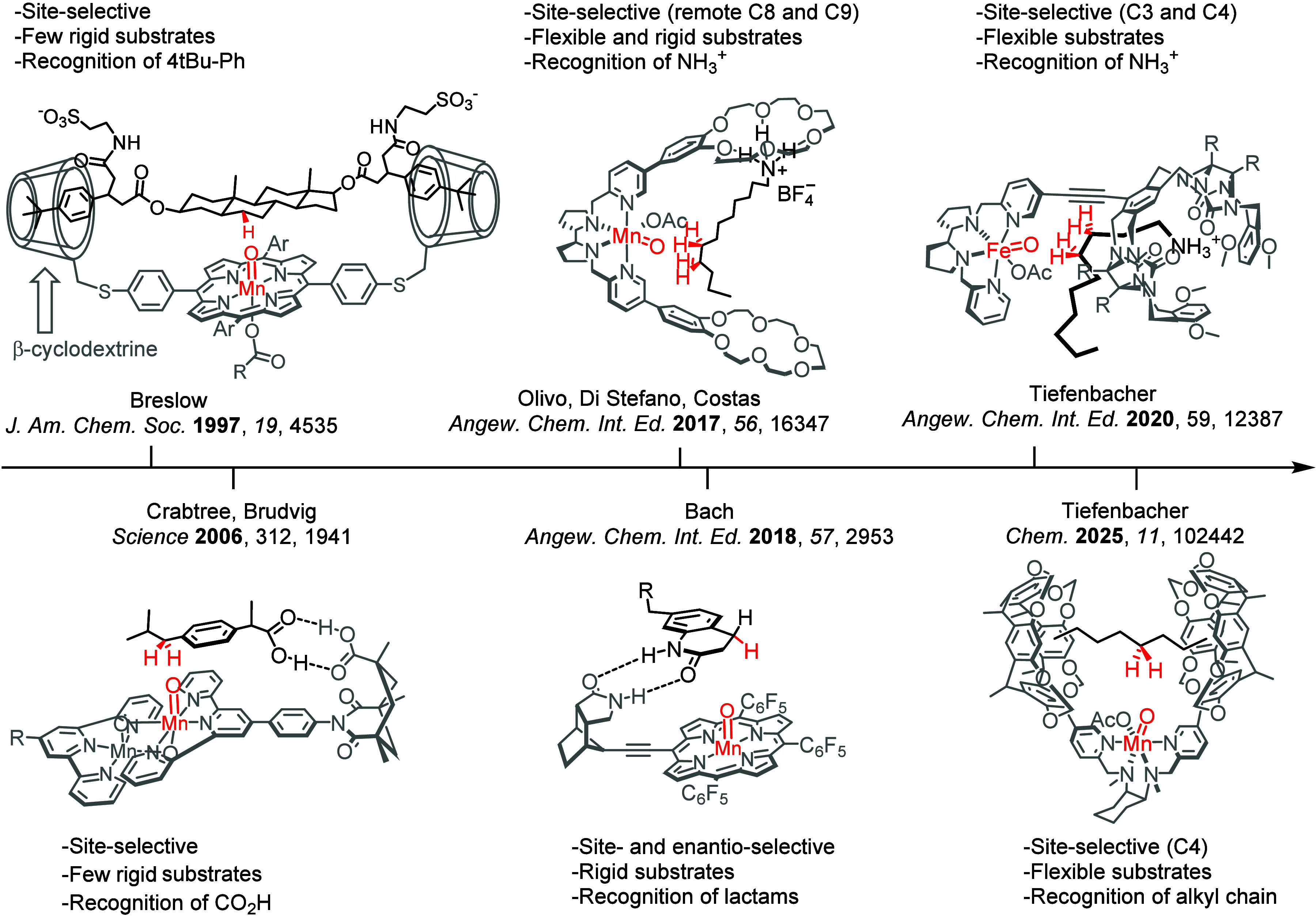
Schematic representation of supramolecular Fe and Mn oxidation catalysts.

In the past decade, different catalyst designs that rely on nonheme Fe or Mn complexes as the catalytic site and crown ether, tweezer, or cavitands as the recognition site proved more tolerant in terms of substrate structure and more versatile in terms of reactivity. Besides our work,^1–4,13–15^ Tiefenbacher reported amplification of C3 and C4 site-selectivity in the oxidation of analogous linear primary ammonium ions upon binding to a tweezer (from 2–19% to 21–51% sel. in spite of their electronic deactivation).^16,17^ More recently, the same group designed a supramolecular catalyst equipped with two cavitand receptors on pyridines β positions able to bind alkanes via solvophobic effect in fluorinated alcohols.^18^ In this case, the substrates are simple, unfunctionalized linear alkanes devoid of functional handles or deactivating groups, and the recognition requires both receptors. Oxidation of linear C7–C11 alkyl chains (and cyclic menthane) preferentially occurs at the ω-4 or ω-5 methylene position (from 17–32% to 52–79% sel., 40–55% total yields).

Bach’s group designed a chiral U-turn lactam receptor that places a single prochiral face of the molecule in proximity of the catalytic site, which is a Mn porphyrin complex. This system enables highly enantioselective oxidation (87–99% ee, 22–68% yields) of rigid lactam substrates at benzylic C–H bonds,^19^ thioethers,^43^ or olefins,^44^ but no oxidation of inactivated C–H bonds has been reported. Remarkably, when there are multiple competing sites, the oxidation occurs close to the recognition point (lactam ring). In addition, there are also some other examples of recognition-guided C–H oxidation that rely on Ru-based catalysts instead of Fe and Mn ones.^45^

## 7 Conclusion and Outlook

In the previous sections, we described the logical flow of information underpinning the rational design of supramolecular oxidation catalysts. The efficiency of this design (structural arrangement of the selected catalytic site, recognition site, and spacer) can be assessed using physical–organic chemistry tools such as kinetic and EM analysis.

Albeit promising, the combination of supramolecular chemistry and catalysis is still in its infancy. In fact, the difficulties met in the design and realization of an efficient supramolecular catalyst often discourage synthetic chemists from taking this path, which requires experimental and intellectual effort, patience against frustrations, and certainly nonimmediate results. However, supramolecular catalysis sometimes represents the most suitable solution to complex synthetic problems such as the selective and predictable functionalization of truly remote sites. This unique potential stems from the peculiar way that reactivity and selectivity are altered. In supramolecular catalysts, the reactivity of the selected site(s) increases more than that of the others by recognition-induced proximity, achieving selectivity in a positive modality. Conversely, in conventional catalysts, selectivity is achieved in a negative modality, with one or more sites put in conditions to be less reactive than the selected one, i.e., via steric repulsion.

Along these lines, supramolecular oxidation catalysts disclosed novel reactivity and selectivity for C–H oxidation, ranging from remote oxidations to the replication of elusive aspects of enzymatic reactivity. However, several issues remain to be addressed. First, the structures of these supramolecular catalysts need to be simplified, as their preparation often represents a significant bottleneck. Their design can still be modulated to bind different groups, improve current precision, target different sites, and address other chemoselectivity and stereoselectivity problems. Eventually, expanding such a HAT/rebound reaction manifold beyond C–H oxidation can disclose new routes for selective C–H functionalization.
